# The Effect of Pentoxifylline on *bcl-2* Gene Expression Changes in Hippocampus after Long-term use of Ecstasy in Wistar Rats 

**Published:** 2013

**Authors:** Zeinab Khazaei Koohpar, Mehrdad Hashemi, Reza Mahdian, Kazem Parivar

**Affiliations:** a *Department of Biology, Science and Research Branch, Islamic Azad University, Tehran, Iran.*; b *Department of Genetics, Islamic Azad University, Tehran Medical Branch, Tehran, Iran.*; c *Biotechnology Research Center, Molecular Medicine Department Pasteur Institute of Iran, Tehran, Iran. *

**Keywords:** Ecstasy, Hippocampus, Pentoxifylline, bcl_2_, Real- time PCR

## Abstract

3,4- Methylenedioxymethamphetamine (MDMA or “Ecstasy”) is a psychoactive and hallucinogenic drug of abuse. MDMA has been shown to produce neurotoxicity both in animals and humans. Recently, the vasodilator drugs such as pentoxifylline is one of the new strategies which have been considered as neuroprotector. In this study effect of pentoxifylline on *bcl- 2 *gene expression changes in hippocampus of rat following long- term use of ecstasy was investigated.

30 male Wistar rats weighing 250-300 g were randomly divided into 5 groups: control (normal), sham (MDMA injection), experimental 1 (MDMA and then PTX injections), experimental 2 (PTX injection and after 1 week, MDMA injection) and vehicle (saline injection) groups. All drugs were injected intraperitoneally.Two weeks later, the hippocampi were removed for studying the changes in *bcl-2 *gene expression. We used quantitative real time PCR for detection of *bcl-2 *gene expression in treated groups and then compared them to control samples. The results showed the gene dosage ratio of 0.49, 0.78 and 1.17 for sham, experimental 1 and experimental 2 groups, respectively. The results also showed the *bcl-2 *gene expression declined in sham group as compared to the experimentalgroups. Furthermore, we observed a significant difference in the *bcl-2 *gene expression between sham and experimental 2 groups. We conclude that quantitative real time PCR could be used as a direct method for the detection of *bcl-2 *gene expression in tested and normal samples.

## Introduction

3,4-Methylenedioxymethamphetamine (MDMA or “Ecstasy”) is a psychoactive recreational hallucinogenic substance and a major drug of abuse worldwide (1, 2). MDMA is known to inhibit the DA Transporter (DAT), NE Trasporter (NET), and 5-HT Transporter (5- HTT) (2). 

MDMA elicit 5-HT, DA and NE release in the brain (2, 3). Neurochemical and anatomical studies have shown that MDMA decreased number of 5-HTT neurons in the rodent neocortex, striatum, and hippocampus (1). The studies have shown that MDMA decrease brain 5-HT transporters in human (4). MDMA has been shown to produce neurotoxicity both in animals and humans (2, 5). Despite more than two decades of studies on MDMA neurotoxic effects, the underlying mechanisms of neurotoxicity still remain to be fully elucidated (2). MDMA and other amphetamines induce serotonergic and dopaminergic terminal neurotoxicity and also neurodegeneration in areas including the cortex, hippocampus, striatum and thalamus (2, 4, 5).Amphetamine and amphetamine derivatives induce apoptosis upon acute and repeated exposures. Apoptotic pathways induced by amphetamine and methamphetamine in neurons seem to be mainly mediated by the mitochondrial apoptotic pathway, associated with a decrease in Bcl-2 levels and direct interference with mitochondrial transmembrane potential (6). Apoptosis is accompanied by endonucleosomal DNA cleavage, activation of caspase-3 and proapoptoic genes (1, 2, 4). It is well known that ecstasy causes apoptosis in brain and liver (7). Direct MDMA 5-HT_2A_ – receptor stimulation produces intracellular oxidative stress that leads to neuronal apoptosis accompanied by caspase-3 activation (5). MDMA has also been shown to cause apoptotic cell death in two different studies using cell cultures (8). Recently, the vasodilator drugs such as pentoxifylline is one of the new strategies which have been considered as neuroprotector (2). Pentoxifylline is a methylxanthine derivative that has multiple properties as anti-inflammatory, inhibitors of free radical production, neuroprotectors, vasodilators, immunomodulators and antiplatelet agents (9, 10-13).

A study has shown that PTX significantly reduced apoptosis of cortical cells following burn injury(9),however,another study indicated that pentoxifylline is able to reduce the severity of lesions in the hippocampus following long-term use of MDMA (14). Pentoxifylline improves learning and memory in glutamate- lesioned rats andboth pentoxifylline and propentofylline reduce neural damage following ischemia (11). In this study, we designed and optimized quantitative real- time PCR assay based on SYBR Green I chemistry to determine the effect of PTX on *bcl-2 *gene expression changes in hippocampus after long-term use of ecstasy in rat.

## Experimental

30 male Wistar rats weighing 250-300g were used in this study. Animals were housed at temperature 22±2 °C and light- controlled environment, with free access to food and water. The rats were divided into five groups, each consisting of n = 6; I: Control group, II: Sham group that on day one rats were treated with a total three intraperitoneal (IP) injections of MDMA (7.5 mg/kg) at 2 h intervals. III: Experimental 1 group that received three IP injection every 2 h, with the last injection of MDMA, pentoxifylline (100 mg/kg)was injected intraperitoneally. IV: Experimental 2 group that rats were injected (IP) with one 100 mg/kg dose of pentoxdifylline at a time, and after 1 week received three IP injections of MDMA (7.5 mg/kg) at 2 h intervals. V: Vehicle group that received saline. (14) 14 days later, the animals were anaesthetized and immediately killed by decapitation. Brains were immediately removed, rinsed with ice cold PBS and hippocampi were rapidly dissected, snap- frozen in liquid nitrogen, and frozen at –80 °C until used for studying the changes in *bcl-2 *gene expression.

*RNA isolation and reverse transcription*

The tissue samples were treated with total RNA isolation reagent (Sigma) as recommended by the manufacturer and the extracted RNA was purified using RNeasy Mini Kit (Qiagen). The concentration and purity of the purified RNA were determined by spectrophotometry. High quality RNAs (A260/280≥1.8) were selected and kept at -80 °C until use for cDNA synthesis. Up to 1 μg RNA was converted to cDNA using QuanTitect® Reverse Transcription Kit (Qiagen) according to the manufacturer›s instructions. To verify the integrity of the cDNA, a PCR experiment was performed using glyceraldehydes-3-phosphate dehydrogenase (GAPDH) specific primer. The primers for real-time PCR of *bcl-2 *and *GAPDH *genes were designed by the Primer Express v.3.0 software (Applied Biosystems, Foster City, USA).

*Real-time PCR with SYBR green I*

The selected primers underwent an extensive search using BLAST tool (www.ncbi.nlm.nih.gov/blast).The characteristics of the primers used in this study have been summarized in Table 1.

**Table 1:** Characteristics of the primers used in the real-time PCR assay

rat-*bcl*-*2*F	ATCGCTCTGTGGATGACTGAGTAC
rat-*bcl*-*2*R	AGAGACAGCCAGGAGAAATCAAAC
rat-*GAPDH*-F	AAGTTCAACGGCACAGTCAAGG
rat-*GAPDH*-R	CATACTCAGCACCAGCATCACC

Real-time PCR was carried out in optical grade 96-well plates (Micro amp, Applied Bio systems, Singapore) at reaction volume of 25 Microlitr , including 12.5 SYBR Green Master Mix (primer design), 300 nM primer and 5 ng template DNA. All samples were run in duplicate. Thermal cycling was performed on the Applied Biosystems 7300 real-time PCR system using the following cycling conditions: 95 °C for 10 min, and 40 cycles at 95 °C for 15 sec, and 60°C for 1 min. Each complete amplification stage was followed by a dissociation stage at 95 °C for 15 sec and 60 °C for 30 sec. Then, temperature was ramped up from 60°C to 95 °C ( 0.03 °C/sec), and fluorescence intensity data was collected continuously over the ramping stage for 20 min. Melting curve analysis was performed according to the dissociation stage data and reactions with a single peak at expected melting temperature (Tm) were considered for further analysis.

*Data analysis*

Quantitative analysis was performed by the measurement of threshold cycle(CT) values during the exponential phase of amplification. The parameter CT was defined as the cycle number at which the amplification plot passed a fixed threshold. In each assay, mCT was the mean CT value of duplicate amplifications. Relative quantity of *bcl-2 *gene was determined using comparative CT method and ΔCT was calculated as the difference between the CT values of the *bcl-2 *and the CT value of *GAPDH *gene. The data were analyzed using the following formula: Gene dosage ratio = 2^−ΔΔCT^, where−ΔΔCT =[mCT*bcl-2 *(test sample)–mCT*GAPDH *gene (test sample) -[mCT*bcl-2 *(normal sample)−mCT*GAPDH *gene (normal sample)]. Gene dosage ratios were relative to the mean ΔCT value of these samples. Data processing was analyzed with ABI Prism 7300 Sequence Detection System (version 1.2.3, Applied Biosystems, UK). The graph preparation were performed using Microsoft Excel 2007 and RJS Graph 3.90.10 (15).

*Statistical analysis*

The results were analyzed with ANOVA test. Analysis of differences between treatment groups followed by post hoc comparsion. A probability level of p < 0.05 was considered significant.

## Results

**Figure 1:**
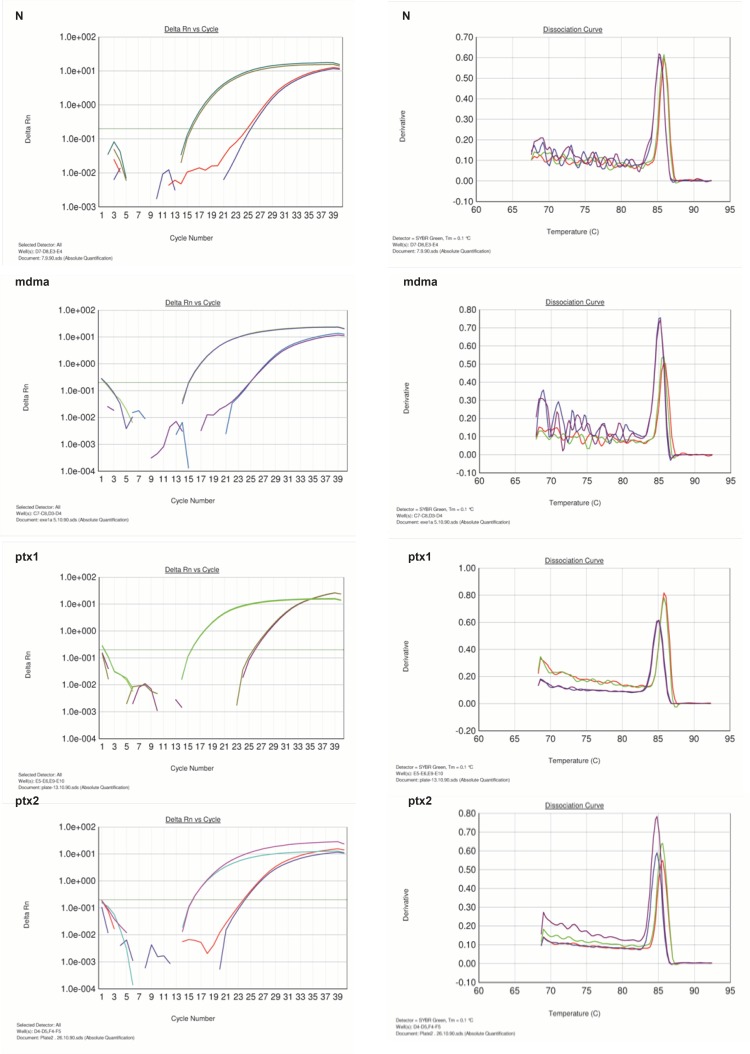
Amplification and melting curve analysis of real-time PCR for *bcl-2*and*GAPDH *genes. N: normal group, MDMA: sham group, PTX1: experimental 1, PTX2: experimental 2

In this study, pentoxifylline effects on *bcl-2 *gene expression changes in hippocampus after long-term use of ecstasy were compared in sham group. Using this method, tested and normal samples were analyzed. As expected, there was a significant difference between the tested and normal samples in real time PCR. To optimize and validate the real-time PCR assay before using ΔΔCT method for gene expression, a validation experiment was performed to determine the PCR efficiencies of the target and the reference genes.The input amount of template DNA was plotted against the corresponding CT values.The slope of the best-fit lines were within the acceptable range of −3.6<slope<−3.1 (http://www.gene-quantification.de). The consistency of all the PCR reactions through a wide range of template DNA concentrations (3–50 ng) was assessed by plotting ΔCT values of *bcl-2 *gene against the input amount of DNA. The absolute slopes of the best-fit lines were ≤0.1 for *bcl-2 *gene which indicated the validity of the ΔΔCT relative quantitation. Melting curve analysis was performed for every single reaction to exclude amplification of non-specific products (Figure 1).

**Figure 2:**
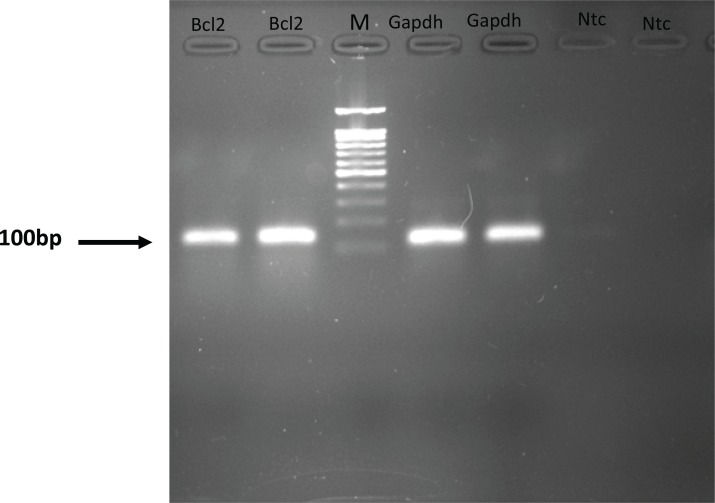
Gel electrophoresis analysis of the PCR products.NTC, non-template control. M, DNA Size marker

 Each valid amplification reaction displayed a single peak at expected Tm. Furthermore, gel electrophoresis analysis of the PCR products revealed a single band with the expected size for each amplicon, (Figure 2). The results showed the gene dosage ratio of 0.49, 0.78 and 1.17 for sham, experimental 1 and 2, respectively. The results also showed the *bcl-2 *gene expression declined in sham group as compared to the experimental groups.Therefore, this study was undertaken to reveal the effect of the pentoxifylline on *bcl-2 *gene expression changes in hippocampus after long- term use of ecstasy in rats. 

## Discussion

(ecstasy) MDMA is a synthetic amphatemine derivative (7). That has been shown to produce neurotoxicity both in animals and humans (2, 5). It is well known that ecstasy causes apoptosis in brain and liver (7). Proteins belonging to the Bcl-2 family seem to play a key role in the apoptotic response triggered by derivatives of amphetamines (16). Bcl-2 family proteins are classically divided into death- inhibiting or death-inducting members.Bcl-2, Bclw and Bclx_L_ are known to enhance cell survival whereas Bax,Bak, Bad and Bid are inducers of death (17).

Bcl_2_ inhibits cytochrome c release from mitochondria elicited by pro-apoptotic Bax, resulting in inhibition of caspase activation and apoptotic death (18).The aim of this study was the effect of pentoxifylline on *bcl-2 *gene expression changes in hippocampus after long-term use of ectasy in Wistar rats. We designed and optimized quantitative real-time PCR assay using SYBR green I technologies from Applied Biosystems. Overexpression of *bcl-2 *in transgenic mice protected neurons from naturally occurring cell death and experimental ischemia (18). METH induces significant increases in the pro-death Bcl-2 family genes Bad, Bax and Bid, and decreases in the anti-death genes Bcl-2 and Bcl-X_L_(17,19).

Moreover, a marked induction of caspase-3 activity was demonstrated in the hippocampus of rats previously administrated with a neurotoxic does of MDMA (20).

METH treatment down-regulated Bcl-2 in the striatum in mice (21). D- amphetamine increases basal apoptosis of neoplastic liver lesions through dysregulation of *bcl-2 *family genes (16); and increased expression of *bcl-2 *can prevent apoptosis of immortalized neuron cells by methamphetamine (22). The results of SYBR Green method were similar to those of obtained from TUNEL assay (14). In the present study, the mean value of the ratios, obtained from tested and normal samples using SYBR Green assay for *bcl-2 *gene, was in agreement with the results reported by Imam *et al. *(21), Tamburini *et al. *(20), Sharifi *et al. *(14) and Sari *et al*. (23).

## Conclusion

This work represents the first systematic study of the simultaneous changes in *bcl-2 *gene expression after long-term use of ecstasy in Wistar rats using real-time PCR technique. Although many studies have focused on *bcl-2 *mRNA expression in amphetamines-induced brain injury, conventional techniques such as Northern blot analysis, quantitative RT-PCR and *in-situ *hybridization are not able to detect small amounts of mRNA, and do not provide quantitative measure of the amounts of mRNA present in the samples. Since the real-time PCR requires a very small amount of mRNA. In the present study, *bcl-2 *mRNA expression significantly declined in sham group as compared to experimental 2 group. These findings are consistent with anti-apoptotic properties of *bcl-2 *gene. Furthermore, this method provides a powerful tool for investigators to study MDMA- induced brain damage and response to treatment drugs with anti-apoptotic agents.

## References

[1] Cadet JL, Krasnova IN, Jayanthi S, Lyles J (2007). Neurotoxicity of Substituted Amphetamines: Molecular and cellular Mechanisms. Neurotoxicity Res.

[2] Capela JP, Carmo H, Remiao F, Bastos ML, Meisel A, Carvalho F (2009). Molecular and cellular mechanisms of ecstasy- induced neurotoxicity: An Overview. Mol. Neurobiol.

[3] Gudelsky GA, Yamamoto BK (2008). Actions of 3.4- methylenedioxymethamphetamine (MDMA) on cerebral dopaminergic, serotonergic and cholinergic neurons. Pharmacology, Biochemistry and Behavior.

[4] Stumm G, Schlegel J, Schafer T, Wurz C, Mennel HD, Krieg JC (1999). Amphetamines induce apoptiosis and regulation of bclX splice variants in neocortical neurons. FASEB J.

[5] Capela JP, Fernandes E, Remiaa F, Bastos ML, Meisel A, Carvalho F (2007). Ecstasy induces apoptosis via 5-HT2A- receptor stimulation in cortical neurons. Neurotoxicology.

[6] Cunha- Oliveira T, Rego AC, Oliveira CR (2008). Cellular and molecular mechanisms involved in the neurotoxicity of opioid and psychostimulant drugs. Brain Res. Reviews.

[7] Miranda M, Bosch-Morell F, Johnsen-Soriano S, Barcia J, Almansa I, Asensio S, Araiz J, Messeguer A, Romero FJ (2007). Oxidative stress in Rat Retina and Hippocampus after chronic MDMA (‘ecstasy’) Administration. Neurochem Res.

[8] Thiriet N, Ladenheim B, Mc Coy MT, cadet JL (2002). Analysis of Ecstasy (MDMA)- induced transcriptional responses in the rat cortex. FASEB J.

[9] Qing J, HongbinJ, Haibin D, WeiyanL, Zhang L (2010). Protective effects of pentoxifylline on the brain following remote burn injury. Burns.

[10] Bruno Rde B, Marques TF, Batista TM, Lima JC, De Arruda KG, Lima PF, Santos Nda S, Cunha GM, Vitor HV, Viana GS (2009). Pentoxifylline treatment improves neurological and neurochemical deficits in rat subjected to transient brain ischemia. Brain Research.

[11] Banfi C, Sironi L, De Simoni G, Gelosa P, Barcella S, Perego C, Gianazza E, Guerrini U, Tremoli E, Mussoni L (2004). Pentoxifylline prevents spontaneous Brain Ischemia in stroke – prone Rats. JPET.

[12] Cunha GMA, Bezerra PJP, Saldanda MDD, Cavalcante MC, Bruin VMS, Viana GSB (2000). Pentoxifylline Improves Learning and Memory in Glutamate- Lesioned Rats. Pharmacology Biochemistry and Behavior.

[13] Baumgartner T (2007). Pentoxifylline. J. Exotic Pet Medicine.

[14] Sharifi ZN, Movassaghi S, Foroumadi A, Hashemi M, Jafari Semnani S, Atashi M (2011). The study of inhibitoryeffect of pentoxifylline on apoptosis ofmale Wistar rat hippocampus after long-termuse of ecstasy. Quarterly J. Developmental Biology.

[15] Kamyar AR, Mahdian R (2010). Development and application of real time quantitative polymerase chain reaction technique using SYBR green 1 in the diagnosis of down syndrome. Molecular Medicine Department Pasteur.

[16] Montiel-Duarte C, Varela-Rey M, Oses-Prieto JA, Lopez-Zabalza MJ, Beitia G, Cenarruzabeitia E, Irabura MJ (2002). 3,4-Methylenedioxymethamphe- tamine (“Esctasy”) induces apoptosis of cultured rat liver cells. Biochimicaet Biophysica Acta.

[17] Cadet JL, Jayanthi S, Deng X (2005). Methamphetamine- induced neuronal apoptosis involves the activation of Multiple Death Pathways. Review. Neurotoxicity Res.

[18] He J, Xu H, Yang Y, Zhang X, Li X-M (2004). Neuroprotective effects of olanzapine on methamphetamine induced neurotoxicity are associated with an inhibition of hyperthermia and prevention of Bcl2 decrease in rats. Brain Res.

[19] Jayanthi S, Deng X, Bordelon M, Mccoy MT, Cadet JL (2001). Methamphetamine causes differential regulation of pro-death and anti-death Bcl2 – genes in the mouse neocortex. FASEB.

[20] Tamburini I, Blandini F, Gesi M, Frenzilli G, Nigro M, Giusiani M, Paparelli A, Fornai F (2006). MDMA induces caspase-3 activation in the limbic system but not in striatum. Ann. N.Y. Acad. Sci.

[21] Imam SZ, Itzhak Y, Cadet JL, Islam F, Jr WS, Ali SF (2001). Methamphetamine- induced alteration in striatal P53 and Bcl2 expressions in mice. Molecular Brain Res.

[22] Cadet JL, Ordonez SV, Ordonez JV (1997). Metham phetamine induces apoptosis in immortalized neural cells: protection by the proto- oncogene, Bcl2. Cynapse.

[23] Sari S, Hashemi M, Mahdian R, Parivar K, Rezayat M (2013). The efect of pentoxifylline on bcl-2 gene expression changes in hippocampus after ischemia-reperfusion in wistar rats by a quantitative RT-PCR method. Iran. J. Pharm. Res.

